# High throughput method for measuring urease activity in soil

**DOI:** 10.1016/j.soilbio.2019.03.014

**Published:** 2019-07

**Authors:** Irene Cordero, Helen Snell, Richard D. Bardgett

**Affiliations:** School of Earth and Environmental Sciences, Michael Smith Building, The University of Manchester, Oxford Road, Manchester, M13 9PT, United Kingdom

**Keywords:** Soil enzymes, Urea amidohydrolase, Nitrogen cycle, Ammonium, Microplate method

## Abstract

Extracellular enzymes break down soil organic matter into smaller compounds and their measurement has proved to be a powerful tool to evaluate the functionality of soils. Urease is the enzyme that degrades urea and is widely considered to be a good proxy of nitrogen (N) mineralisation. But the methods available to measure this enzyme are time consuming; as such, urease is not commonly included in standard enzyme profiling of soils. We developed a fast, high throughput and reproducible colorimetric microplate technique to evaluate urease activity in soil. The method involves the incubation of soil slurries in 96-deepwell blocks with urea solutions and the measurement, by colorimetric reaction, of ammonium produced. We compared the new method with existing methods, yielding comparable results, and evaluated optimal conditions for urease analysis (soil slurry concentration, substrate concentration, incubation times and extractant salt concentration) in different grassland soils. The method proved to be a faster, higher throughput, and more precise alternative to existing methods for evaluating this important N-related enzyme.

## Highlights

• A new method was designed to measure urease activity in soil.
• The method is fast, high throughput, and reproducible.
• The new method proved to be a faster and more precise alternative to existing methods.

## 1 Introduction

Organic matter decomposition is a complex process that requires the combined action of multiple organisms. In particular, soil microorganisms produce and release a myriad of different extracellular enzymes that help to depolymerise and mineralise complex organic compounds into smaller molecules that can then be assimilated (Burns et al., 2013). The assessment of different soil extracellular enzymes has proved to be a powerful tool for evaluating the functionality of soils in the context of nutrient cycling and microbial nutrient demand (Sinsabaugh and Follstad Shah, 2012). Among the N-related extracellular enzymes, urease (urea amidohydrolase, EC 3.5.1.5) is the enzyme involved in the degradation of urea. In general, amidohydrolases are a good proxy for N mineralisation, as they transform small N-containing organic substrates into inorganic N compounds such as ammonia (Sinsabaugh and Follstad Shah, 2012). Specifically, urease catalyses the breakdown of urea into NH_3_ and CO_2_:

NH_2_CONH_2_ + H_2_O → CO_2_ + 2NH_3_

Urease is widely distributed in soils and was one of the first soil enzymes to be experimentally evaluated (Conrad, 1940). Since that time, a range of methods have been proposed to measure urease (Tabatabai, 1994). Its significance resides in the: (1) important role of this enzyme in the N cycle, generating accessible N for plant growth, and (2) widespread use of urea as a fertilizer (Sinsabaugh and Follstad Shah, 2012, Tabatabai, 1994). The most popular method to measure urease, still widely used today, was developed in 1988 by Kandeler and Gerber. This method involves the incubation of samples in large containers, such as 100 ml Erlenmeyer flasks, which is more time consuming than high throughput methods that are now commonly used for the evaluation of other soil enzymes such as *β*-glucoside or N-Acetyl-*β*-D-glucosaminidase (Marx et al., 2001). For example, to evaluate 45 samples for urease activity takes approximately a full day, or even two, using the method of Kandeler and Gerber (1988), while high throughput methods can evaluate the same number of samples in a few hours. As such, this important N-related enzyme assay is often omitted from studies that use high-throughput enzyme methods to evaluate organic matter decomposition capacity of soils (see for example Crowther et al., 2015, Razavi et al., 2017, Schimel et al., 2017, Sinsabaugh et al., 2008).

Here, we describe a fast, high throughput method to evaluate urease in soils, which we hope will facilitate the measurement of this important enzyme alongside other enzymes. Specially, we compare our new plate method with the original method of Kandeler and Gerber (1988), and check the yield and reproducibility of the method in different grassland soils. We also optimised the different parameters of the enzymatic reaction, such as linearity or substrate saturation, which are crucial for an accurate measurement of the potential enzymatic activity. We note that this is not the first attempt to develop a high throughput urease assay. A microplate assay to measure urease was published by Sinsabaugh et al. (2000), which is based on the incubation of very low concentrated soil suspensions with urea in 96-well plates and the evaluation of the ammonia produced directly in the soil suspensions, avoiding centrifugation steps. However, the method has not been commonly included in the standard enzyme profiling methods. The reason for this could be the considerable analytical variation of the method; as such, it requires large numbers of analytical replicates (up to 16), which limits the number of samples that can be processed per plate. The method that we present here is also based on the incubation of soil suspensions with urea in 96-well plates. However, as a direct adaptation of the method of Kandeler and Gerber (1988), it includes an extraction step with KCl and a centrifugation step before ammonia detection, albeit in smaller containers. Here, our goal was to test a new method to evaluate urease activity in soils, compare it with existing methods, and optimise the reaction conditions for different grassland soils.

## 2 Materials and methods

### 2.1 Soil collection and characterization

Soils from a wide range of grassland types were collected in the UK and Austria (Table 1) from the surface 10 cm (top soil). We focussed on grasslands because they are a widespread ecosystem type, covering 40% of the agricultural area in Europe and 65% in the UK (O'Mara, 2012). Further, given that most grasslands are grazed by livestock and other grazing animals, they receive high inputs of urea from urine, which can have a significant impact on urease activity of soils (McNaughton et al., 1997). Soil samples were sieved to 4 mm and kept at 4 °C until analysis (maximum storage time 4 weeks). Storage time can affect urease activity, although this effect is enzyme and soil type dependent (Lee et al., 2007, Pancholy and Rice, 1972). Therefore, we recommend a shorter storage time, if feasible. Soil pH was measured at a soil/water ratio of 1:2.5 (w:v), soil water content (SWC) was evaluated gravimetrically, and organic matter (OM) content was evaluated by loss on ignition, following standard procedures (Sparks, 1996).

**Table 1:** Details of the collection area and chemical characteristics of the soils used in this experiment. SWC: soil water content, OM: organic matter content.

Soil Name	Location	Latitude	Longitude	Elevation (m asl)	Management regime^a^	Soil type	pH	SWC (wet weight)	OM (%)
Wardlow Hay Cop	Wardlow, Peak District, UK	53° 15′ 42.20″ N	1° 43′ 58.80″ W	350	Extensive	Freely drained, slightly acid cambisol^b^	6.79	47%	20.7
Selside	Selside Shaw, Yorkshire Dales, UK	54° 10′ 47.33″ N	2° 20′ 9.39″ W	301	Extensive	Acidic loamy cambisol with wet peaty surface horizon^b^	5.83	42%	11.0
Sharp Int	Ivescar, Chapel-le-Dale, Yorkshire Dales, UK	54° 12′ 45.72″ N	2° 23′ 27.97″ W	324	Intensive	Acid stagnosol with a peaty surface horizon^b^	6.37	59%	18.7
Sharp Ext	Ivescar, Chapel-le-Dale, Yorkshire Dales, UK	54° 12′ 45.53″ N	2° 23′ 26.44″ W	320	Extensive	Acid stagnosol with a peaty surface horizon^b^	5.75	46%	12.4
River	Winterscales, Chapel-le-Dale, Yorkshire Dales, UK	54° 13′ 1.88″ N	2° 22′ 39.16″ W	318	Extensive	Acid stagnosol with a peaty surface horizon^b^	6.00	34%	8.8
Hill	Winterscales, Chapel-le-Dale, Yorkshire Dales, UK	54° 12′ 59.10″ N	2° 22′ 38.72″ W	323	Extensive	Blanket peat (histosol)^b^	4.75	83%	75.4
Vent	Vent, Tyrol, Austria	46° 51′ 48.84″ N	10° 53′ 54.78″ E	2450	Extensive in summer	Dystric lithosols^c^	4.63	53%	28.1
HM2.5, HM2.12, HM3.17	Hohe Mut, Tyrol, Austria	46° 50′ 55.38″ N	11° 01′ 48.17″ E	2560	Extensive in summer	Dystric lithosols^c^	5.43 5.51 5.40	41% 32% 37%	18.0 11.0 17.0
Dale 2	Yorkshire Dales, UK	54°11′33.75″ N	2°20′49.10″ W	348	Extensive	Acidic loamy leptosol with a peaty surface horizon^b^	4.26	50%	14.4

asl: above sea level.

a Intensive management refers to agriculturally improved grassland, typically with fertilizer addition of >100 kg N ha^−1^ yr^−1^, high livestock stocking rates, and frequent cuts for hay and/or silage. Extensive management refers to fields that have not received inorganic fertilizers and are grazed at low livestock densities, with, in some cases, an annual cut for hay.

b Based on the UK soil observatory (Cranfield University, 2019).

c Based on the Soil Map of Austria (Rieck, 1989).

### 2.2 New high throughput plates method

For each grassland, 4 g soil was suspended in 10 ml of sodium acetate buffer (50 mM, pH 5.0) in conical flasks to create a slurry. A common pH was selected for all the samples for comparison purposes. However, if working with samples with a different pH range, the pH of the buffer solution can be adjust to better represent the real conditions in the field (*in situ* approach, Burns et al., 2013). Soil slurries were thoroughly homogenized by stirring until no aggregates were visible, using a spatula when needed to help breakdown aggregates. Ultra-sonication of the slurries at low energy could be done to better expose and release extracellular enzymes immobilized on humic colloids (Marx et al., 2001). From those slurries, 0.25 ml was extracted under continuous stirring and placed into deep-well blocks (2 ml deep). Each sample was dispensed into six wells: four wells to be incubated with urea (analytical replicates), and two wells for controls (no substrate added) to account for the initial ammonia content in the soils. Each well received 0.1 ml of 80 mM urea solution (analytical replicates) or 0.1 ml of buffer (soil controls). Additionally, four substrate controls were added per block, where soil slurry was replaced by buffer to account for a possible colour of the urea solution or any ammonia contamination in the reagents. Blocks were covered with Parafilm and incubated at 18 °C for 2 h under continuous shaking (orbital microplate shaker). After incubation, 1 ml of 2 M KCl was added to each well (to extract available ammonia) and blocks were well covered (with tight plate seals to prevent mixing between wells) and shaken for a further 30 min. Blocks were then centrifuged (2900×*g*, 5 min), and 75 μl of the supernatant were pipetted into transparent 96-well plates and mixed with 75 μl of water. Ammonia concentration was evaluated by Berthelot reaction (Krom, 1980), which was done with two different reagents: an oxidation solution (1 mg ml^−1^ dichloroisocyanuric acid sodium salt dehydrate) and a colour reagent (a mixture 2:1 (v:v) of solution A and B that should be mixed just before use). Solution A: 0.15 M NaOH. Solution B: 170 mg ml^−1^ sodium salicylate and 1.278 mg ml^−1^ sodium nitroprusside dehydrate. For ammonia measurement each well received 75 μl of colour reagent followed by 30 μl of the oxidation solution. Each well was properly mixed by pipetting and colour was evaluated after 30 min by absorbance at 650 nm in a microplate reader (EZ400 Research, Biochrom, Germany). Urease activity (μg N—NH_4_^+^ h^−1^ g^−1^ dw soil) was calculated as:

$$Activity=\;\frac{\Delta NH_{4}^{+}\left(\mu g/ml\right)\times Volume\;in\;well\;\left(1.35\;ml\right)}{Incubation\;time\;\left(2\;h\right)\times g\;wet\;soil\;in\;well\;\left(0.1\;g\right)\;\times \left(1-SWC\right)}$$

$\Delta NH_{4}^{+}$ being the concentration of ammonia in the incubated wells minus the ammonia in soil controls minus the ammonia in substrate controls. These concentrations were calculated by comparison with a standard curve of ammonia (0–3.5 μg ml^−1^) prepared in the same sample matrix (1:0.35:1.35 v:v:v 2 M KCl:acetate buffer:water), and taking into account the dilution factor (2) of the samples after being transferred into the reading plates. The ‘volume in well’ and ‘soil in well’ in the above equation refer respectively to the volume of liquid and the amount of soil in the deep-well block where the soil slurries where incubated. Therefore, the volume in well includes the soil slurry (0.25 ml), the urea solution (0.1 ml) and the KCl (1 ml).

### 2.3 Optimisation procedures

For the plate method, soils were incubated as slurries instead of intact fresh soil (as recommended in the original tubes method), due to the difficulty of dispensing homogenous amounts of fresh soil into wells. Two different soil slurry concentrations were tested: 0.8 g soil ml^−1^ buffer (C1) and 0.4 g soil ml^−1^ buffer (C2), in sodium acetate buffer (50 mM, pH 5.0). Further, optimum conditions of substrate concentration and incubation time were evaluated using seven different soils. Optimum substrate concentration to ensure maximum enzyme activity was checked by incubating the soils with different urea solution concentrations (2.5, 5, 10, 20, 40, 80, 120, 160, 250, 500, 750, 1000, 1250, 1500, 1750 and 2000 mM urea). Measurement of enzymatic activities under non-saturating conditions will underestimate the potential enzyme activity (German et al., 2011). To confirm the linearity of the reaction (as the enzyme activity is calculated as a rate), soils were incubated for several different time periods (0.5, 1, 1.5, 2, 2.5 and 3 h). Finally, a calibration curve with ammonia was carried out with 1 M and 2 M KCl as sample matrix, to evaluate the effect of the salt concentration in the colour reaction.

### 2.4 Original tubes method

The plate method was compared with the original method of Kandeler and Gerber (1988), hereafter called the “tubes method”, in five different soils. Briefly, 1 g soil was mixed with 500 μl of 80 mM urea solution and incubated at 18 °C for 2 h (no shaking). After incubation, soils were extracted with 10 ml of 2 M KCl by horizontally shaking them for 30 min at 300 rpm, centrifuging at 2900×*g* for 5 min and then filtering through Whatman 42 filter paper. The amount of ammonia after incubation (t_1_) was compared with the ammonia present in the soils before the incubation (t_0_). To establish ammonia concentration at t_0_, soils were mixed with 500 μl of the urea and immediately extracted as described above. Ammonia in extracts was evaluated with an Auto Analyzer AA3 (Seal Analytical, UK) by the Berthelot reaction (Krom, 1980).

### 2.5 Statistical analyses

To compare the plate method with the tubes method, data were analysed by one-way ANOVA. Time curves were fitted by linear regression while substrate concentration curves were fitted by non-linear regression to the Michaelis-Menten equation ((*V*_max_ [substrate])/(*K*_*m*_ + [substrate])). All analyses were carried out with R software (R Core Team, 2016).

## 3 Results

### 3.1 Soil characteristics

Soil organic matter content ranged from 8.8% (River) to 75.4% in the organic peat soil (Hill) (Table 1). Soil pH ranged from 4.26 (Dale 2) to 6.79 (Wardlow Hay Cop). These two characteristics can highly affect the enzyme capacity of soils.

### 3.2 Method optimisation

**Fig. 1:**
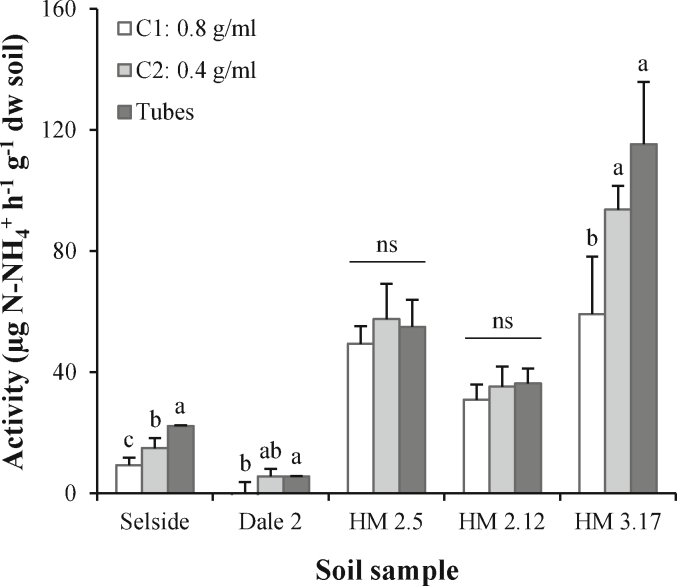
Urease activity measured in plates with two different soil slurry concentrations (C1: 0.8 g soil ml^−1^ buffer and C2: 0.4 g soil ml^−1^ buffer), and in tubes. All samples were incubated for 2 h with 80 mM of urea solution as a substrate. For each soil, different letters denote significant differences among methods following Tukey test (p < 0.05). ns: not significant. Values = mean ± standard deviation, n = 4.

**Fig. 2:**
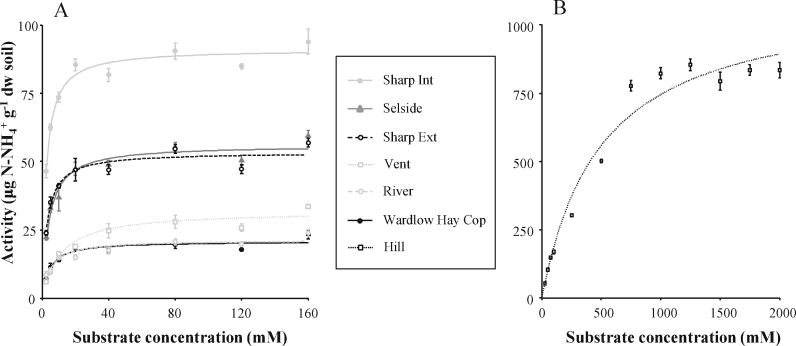
Urease activity as a function of substrate (urea) concentration for all soils except “Hill” (A) and “Hill” sample (B). Lines were fitted with non-linear regression to the Michaelis-Menten equation ((*V*_max_ [substrate])/(*K*_*m*_ + [substrate])). Values = mean ± standard deviation, n = 4.

**Fig. 3:**
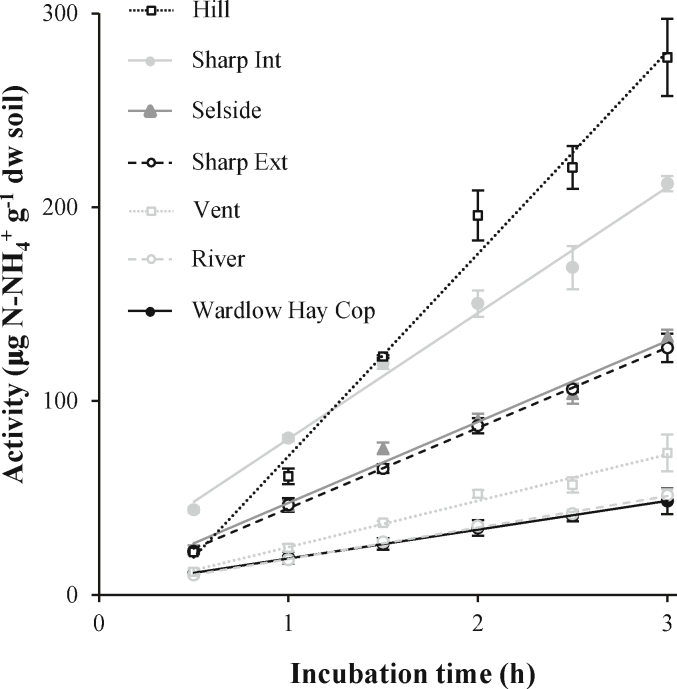
Urease activity over time and linear fit. Values = mean ± standard deviation, n = 4.

**Fig. 4:**
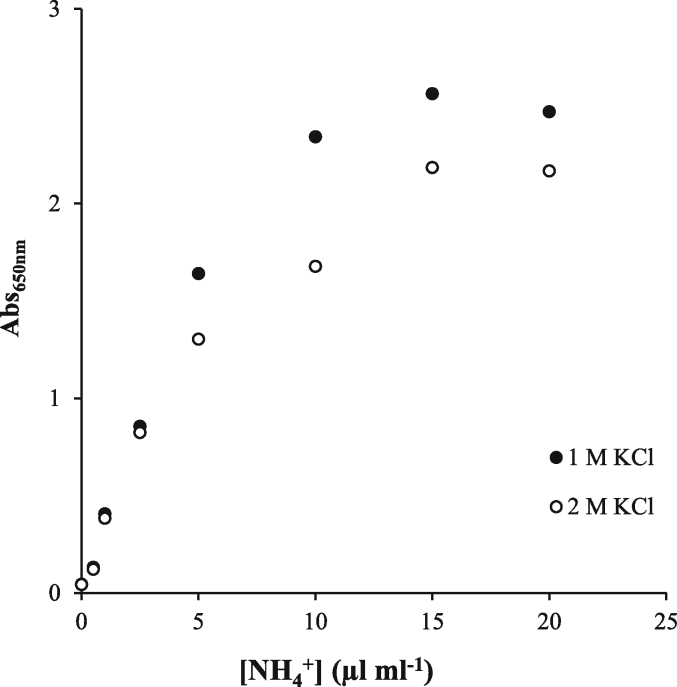
Ammonia calibration curves with different sample matrixes (1 M or 2 M KCl).

At high soil slurry concentration (C1), the plate method measured significantly lower potential enzyme activities than at a low slurry concentration (C2) in three of the five soils (Fig. 1). Most soils showed similar substrate concentration curves (Fig. 2a), where the enzyme activity saturated above 40 mM or 80 mM urea solution. The only exception was soil “Hill” (peat), which saturated at higher concentrations (1000 mM) (Fig. 2b). In all cases, enzyme activity increased linearly over the evaluated incubation time (Fig. 3). Ammonia determination was successfully carried out at 1 M or 2 M KCl concentration (Fig. 4), but the colour reaction saturated earlier with 2 M KCl than with 1 M KCl (Fig. 4).

### 3.3 Methods comparison

The optimised plate method yielded comparable results to the tubes method (Fig. 1) at a slurry concentration of 0.4 g ml^−1^ (C2). However, when incubations were carried out at a higher slurry concentration (C1, 0.8 g ml^−1^), there were significant differences in the 3 out of 5 soils analysed, rendering this concentration unsuitable for measuring urease activity. At a slurry concentration of 0.4 g ml^−1^, the relative differences in potential urease activity between soil samples were highly similar across the plate and tubes methods. For example, urease activity was 6 times greater in HM 2.12 than Dale 2 soil, and this difference was consistent across methods (i.e. 6.29 times higher in the plate method *vs*. 6.45 times higher in the tubes method). Also, we found that HM 2.5 activity was half that of HM 3.17 soil, and this difference was likewise consistent across methods (0.61 times in the plate method *vs*. 0.48 times in the tubes method). There was no significant difference in the potential urease activity measured by the two methods for four out of the five soils tested. With a slurry concentration of 0.4 g ml^−1^, the plate method recorded a lower potential urease activity than the tubes method in one of the five soils tested (Selside). Despite this single soil difference between the methods, both methods ranked the five soils similarly in terms of their potential urease activity.

## 4 Discussion

Overall, our results highlight the suitability of this new plate method to measure potential urease activity in grassland soil. Our proposed plate method yielded comparable results to the original tubes method (Fig. 1). At the lowest soil slurry concentration (0.4 g ml^−1^) tested, there was no difference in urease activity between the plate and tubes method in all soils, but one (Selside); as such, we recommend that this slurry concentration is used for assessment of urease activity in grassland soils. However, at a higher soil slurry concentration (0.8 g ml^−1^), the recorded enzymatic activity was in all cases lower, and significantly different from the tubes method. This is likely attributed to a higher ratio soil-to-buffer, which might have inhibited enzyme dispersion in the solution, resulting in an apparent lower enzymatic activity.

Urease is a hydrolytic enzyme that follows Michaelis-Menten kinetic, i.e., the activity of the enzyme increases as the substrate concentration increases, until it reaches a maximum activity rate (Fig. 2). In general, the evaluation of soil enzymes such as urease aims to measure the maximum enzyme activity, to maximize the power to detect differences between soil samples (German et al., 2011). Thus, the assay should be done at saturating substrate concentrations. We found that most of the grassland soils tested showed similar substrate concentration curves, except the peat soil, which saturated at higher concentrations. This fact is probably related to its extremely high organic matter content, as high organic matter content has been frequently correlated with higher enzymatic capacity of soils (Sinsabaugh et al., 2008). Therefore, we suggest using 80 mM urea solution for most grassland soils, and 1000 mM for peat. However, we strongly recommend checking the substrate saturation curve for each set of soils samples to be assayed, as the saturation point varies widely between soil types and may also vary seasonally within some soils, especially those that are subject to strong seasonality such as alpine soils which are seasonally snow-covered (Lipson et al., 2002, Schmidt et al., 2007).

Another important parameter to take into account when evaluating soil enzymes is the incubation time. As the enzyme activity is calculated as a rate, it is important to confirm that the reaction is linear over time (German et al., 2011). In our test, enzyme activity increased linearly over the evaluated incubation time in all cases (Fig. 3), confirming that the selected incubation time (2 h) is adequate. In the case of soils with very low enzymatic activities (e.g., subsoils or soils of very low organic C content) the detection of very low rates of ammonia production can be challenging. In such cases, we recommend that the incubation time should be prolonged: the rate of ammonia production will be the same, as it increases linearly (at least up to 3 h), but the absolute value of ammonia produced will be higher and hence easier to precisely detect.

Ammonia ions are more efficiently extracted under high salt concentrations (Mulvaney, 1996). Although the ammonia evaluated during the enzyme assay is recently produced, it can quickly bind or adsorb to inorganic minerals depending on soil characteristics. Hence, we recommend extracting the ammonia after enzyme incubation with high salt concentrated (2 M KCl) solutions to accurately evaluate all the ammonia produced. But the colour reaction saturated earlier with 2 M KCl than with 1 M KCl concentration, rendering into lower determination capacity of the method (Fig. 4). The lower colour reaction at higher salt concentrations could be related to a higher buffering capacity that decreases the final pH of the reaction (Hansen and Koroleff, 2007). Therefore, soil extracts should be better analysed diluted to 1 M KCl before colour determination, as suggested in the proposed plate method.

Compared to the original tubes method, the new method presented here offers the main advantage of being high throughput, which enables the evaluation of high number of samples in a short time. Nevertheless, the results are still comparable to the tubes method. Compared to the other plate method published (Sinsabaugh et al., 2000), our new method presents some advantages. It has higher throughput (15 samples per plate in our method compared to 4), a shorter incubation time (2 h *vs* 18 h), smaller variance between analytical replicates (<10% in our method, with a mean value of 3% and 4 replicates, compared to 5–20% with 16 analytical replicates in theirs) and a more accessible measurement of NH_4_^+^ (they use a commercial measuring kit, reagent packets from Hach, while we specify the individual reagents for the reaction). In conclusion, the method presented here represents a fast, high throughput, and reproducible method to measure urease in soils, an important enzyme related to N cycling in soil.

## References

[bib1] Burns R.G., DeForest J.L., Marxsen J., Sinsabaugh R.L., Stromberger M.E., Wallenstein M.D., Weintraub M.N., Zoppini A. (2013). Soil enzymes in a changing environment: current knowledge and future directions. Soil Biology and Biochemistry.

[bib2] Conrad J.P. (1940). Hydrolysis of urea in soils by thermolabile catalysis. Soil Science.

[bib3] Cranfield University (2019). The soils guide. http://www.landis.org.uk/.

[bib4] Crowther T.W., Thomas S.M., Maynard D.S., Baldrian P., Covey K., Frey S.D., van Diepen L.T.A., Bradford M.A. (2015). Biotic interactions mediate soil microbial feedbacks to climate change. Proceedings of the National Academy of Sciences.

[bib5] German D.P., Weintraub M.N., Grandy A.S., Lauber C.L., Rinkes Z.L., Allison S.D. (2011). Optimization of hydrolytic and oxidative enzyme methods for ecosystem studies. Soil Biology and Biochemistry.

[bib6] Hansen H.P., Koroleff F., Grasshoff K., Kremling K., Ehrhardt M. (2007). Determination of nutrients. Methods of Seawater Analysis.

[bib7] Kandeler E., Gerber H. (1988). Short-term assay of soil urease activity using colorimetric determination of ammonium. Biology and Fertility of Soils.

[bib8] Krom M.D. (1980). Spectrophotometric determination of ammonia: a study of a modified Berthelot reaction using salicylate and dichloroisocyanurate. The Analyst.

[bib9] Lee Y.B., Lorenz N., Dick L.K., Dick R.P. (2007). Cold storage and pretreatment incubation effects on soil microbial properties. Soil Science Society of America Journal.

[bib10] Lipson D.A., Schadt C.W., Schmidt S.K. (2002). Changes in soil microbial community structure and function in an alpine dry meadow following spring snow melt. Microbial Ecology.

[bib11] Marx M.C., Wood M., Jarvis S.C. (2001). A microplate fluorimetric assay for the study of enzyme diversity in soils. Soil Biology and Biochemistry.

[bib12] McNaughton S.J., Zuniga G., McNaughton M.M., Banyikwa F.F. (1997). Ecosystem catalysis: soil urease activity and grazing in the Serengeti ecosystem. Oikos.

[bib13] Mulvaney R.L., Sparks D.L. (1996). Nitrogen—inorganic forms. Methods of Soil Analysis Part 3—Chemical Methods.

[bib14] O'Mara F.P. (2012). The role of grasslands in food security and climate change. Annals of Botany.

[bib15] Pancholy S.K., Rice E.L. (1972). Effect of storage conditions on activities of urease, invertase, amylase, and dehydrogenase in soil. Soil Science Society of America Journal.

[bib16] R Core Team (2016). R: a Language and Environment for Statistical Computing.

[bib17] Razavi B.S., Liu S., Kuzyakov Y. (2017). Hot experience for cold-adapted microorganisms: temperature sensitivity of soil enzymes. Soil Biology and Biochemistry.

[bib18] Rieck W. (1989). Bodenkarte von Osterreich.

[bib19] Schimel J., Becerra C.A., Blankinship J. (2017). Estimating decay dynamics for enzyme activities in soils from different ecosystems. Soil Biology and Biochemistry.

[bib20] Schmidt S.K., Costello E.K., Nemergut D.R., Cleveland C.C., Reed S.C., Weintraub M.N., Meyer A.F., Martin A.M. (2007). Biogeochemical consequences of rapid microbial turnover and seasonal succession in soil. Ecology.

[bib21] Sinsabaugh R.L., Follstad Shah J.J. (2012). Ecoenzymatic stoichiometry and ecological theory. Annual Review of Ecology, Evolution, and Systematics.

[bib22] Sinsabaugh R.L., Lauber C.L., Weintraub M.N., Ahmed B., Allison S.D., Crenshaw C., Contosta A.R., Cusack D., Frey S., Gallo M.E., Gartner T.B., Hobbie S.E., Holland K., Keeler B.L., Powers J.S., Stursova M., Takacs-Vesbach C., Waldrop M.P., Wallenstein M.D., Zak D.R., Zeglin L.H. (2008). Stoichiometry of soil enzyme activity at global scale. Ecology Letters.

[bib23] Sinsabaugh R.L., Reynolds H., Long T.M. (2000). Rapid assay for amidohydrolase (urease) activity in environmental samples. Soil Biology and Biochemistry.

[bib24] Sparks D.L. (1996). Methods of Soil Analysis Part 3—Chemical Methods.

[bib25] Tabatabai M.A., Weaver R.W., Angle S., Bottomley P., Bezdicek D., Smith S., Tabatabai A., Wollum A. (1994). Soil enzymes. Methods of Soil Analysis. Part 2 - Microbiological and Biochemical Properties.

